# Representation and generalizability in clinical research: Back to basics

**DOI:** 10.1017/cts.2025.10202

**Published:** 2025-11-28

**Authors:** Shari Messinger, Ann Brearley, Barbara H. Brumbach, Manisha Desai, Felicity T. Enders, Jodi Lapidus, Mary Sammel, Heidi M. Spratt

**Affiliations:** 1 Department of Public Health Science, Division of Biostatistics and Bioinformatics, University of Miami Miller School of Medicine, Miami, FL, USA; 2 Division of Biostatistics and Health Data Science, School of Public Health, University of Minnesota, Minneapolis, MN, USA; 3 Oregon Health and Science University-Portland State University School of Public Health, Portland, OR, USA; 4 Quantitative Sciences Unit, Stanford University School of Medicine, Stanford, CA, USA; 5 Department of Quantitative Health Sciences, Division of Clinical Trials and Biostatistics, Mayo Clinic, Rochester, MN, USA; 6 Department of Biostatistics & Informatics, Colorado School of Public Health, University of Colorado Anschutz Medical Campus, Aurora, CO, USA; 7 Department of Biostatistics and Data Science, University of Texas Medical Branch, Galveston, TX, USA

**Keywords:** Generalizability, representation, external validity, efficiency, statistical bias, heterogeneity of effects

## Introduction

Core principles of statistical inference emphasize that generalizable and reliable conclusions in clinical and translational research depend on study samples that are representative of the populations to whom inference will be applied. Representation is not only a design consideration but a benchmark of scientific credibility and rigor. Statisticians are trained in methods to reduce statistical bias in clinical studies to ensure generalizable and reliable results. Statistical bias undermines external validity and generalizability, both of which are essential to the translation of scientific findings to medical practice. To that end, sampling methods (i.e., stratified random sampling) [1] and research design strategies (i.e., randomized block design) [2] have been developed to mitigate statistical bias from nonrepresentative study groups. When representativeness is not adequately considered, the potential for non-generalizability increases.

**Figure 1.**
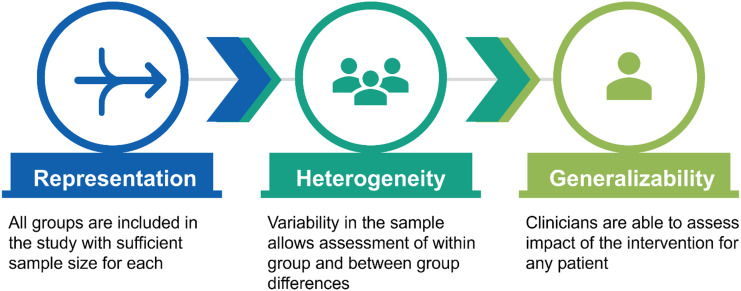
Relationship among representation, heterogeneity, and generalizability in clinical studies. Adequate representation ensures all groups are included with sufficient sample size; heterogeneity enables assessment of within- and between-group differences; and together these support generalizability of findings to individual patients.

With the 2025 changes to the National Institutes of Health (NIH) scientific review process, all reviewers are required to complete two new trainings prior to providing peer review in scientific review panels (commonly called “study sections”) [3]. While the need to ensure clinical research studies avoid statistical bias and maximize generalizability is not new, the emphasis of these topics within the two required trainings is new [4]. We offer the following to aid scientists and clinical researchers in understanding nuance within this topic (Figure 1).

## Ensuring generalizability and external validity

It is well understood that clinical trials have excellent internal validity [5]. However, even when designed well, many trials face issues of external validity, where findings do not generalize to populations of interest. This phenomenon occurs because those eligible for the trial and those who participate differ in meaningful ways from the target population (e.g., consider a study of an intervention to improve quality of life in those who experience joint pain, where only those who experience minimal pain agree to participate in the study). Consequently, trial findings may only be relevant for a subset of the target population. Alternatively, studies conducted with broadly representative participants ensure results will be generalizable to populations of interest, that is, have external validity. For example, consider the Women’s Health Initiative, designed to evaluate the effect of hormone replacement therapy (HRT) on cardiovascular outcomes among postmenopausal women aged 50–79 years [6]. Findings revealed that HRT increased risk of coronary heart disease and stroke [7]. Consequently, controversy still exists today as gynecologists treating women under age 60 do not believe the trial findings apply to this subgroup. Women typically initiate HRT prior to menopause and are younger and healthier than the majority of those enrolled in the trial being older and past menopause [8]. When representation is inadequate, bias can move from abstract to tangible harm. From warfarin dosing errors to pulse oximetry inaccuracies in patients with darker skin, the lack of representative data has repeatedly translated into inadequate care [9,10]. Contemporary studies also illustrate gaps that limit external validity. Many oncology clinical trials under-enroll older adults, despite this group representing most individuals diagnosed with cancer, resulting in treatment evidence potentially non generalizable to those most likely to receive therapy [11]. Similarly, digital health studies frequently recruit younger, technology-engaged participants, limiting applicability to older adults. To ensure findings inform medical practice, it is critical that scientists design and interpret trial findings appropriately for clinical decision-making.

## Accounting for population variability

It is important to include a broad range of individuals in clinical research to understand sources of variability that contribute to differences in health outcomes between subgroups. Thus, it is considered responsible practice to include as many sources of variability as are present in the real-world setting [12,13]. Often variability in outcomes is explained by population subgroup differences, including differences in race, ethnicity, gender, culture, age, geography, etc. Failure to include a sufficiently broad range of participants representative of the target population can lead to findings that do not accurately reflect the population [12,13]. For example, a review of vaccine trials from 2011 to 2020 highlights limited population representation, with nearly half lacking American Indian or Alaska Native participants and over 60 percent lacking Hawaiian or Pacific Islander participants, precluding analysis of subgroup differences [14]. Additionally, failure to accurately account for population variability reduces efficiency and precision in estimating treatment effects and other key relationships of interest [13]. Even when participant representation is adequate, accounting for sources of variability is necessary to increase efficiency, improving precision in estimating treatment effects. In clinical trials, ignoring population heterogeneity can result in both failure to identify harm and failure to discover truly effective treatments [14].

## Understanding heterogeneity of effects

Along related lines, differential treatment or exposure effects often exist across population subgroups. This heterogeneity of effect cannot be evaluated and understood if study samples lack representation [15]. Heterogeneity related to biological differences as well as social and contextual determinants of health (i.e., economic status, geography, access, and environment), which shape “what works for whom, and under what conditions” may affect whether a treatment or exposure will be effective or harmful [15–18]. For example, glucose-6-phosphate dehydrogenase (G6PD) is an enzyme crucial for protecting red blood cells from oxidative damage. People with G6PD deficiency are at risk for hemolytic anemia, which can be triggered by some treatments for malaria prevention, sulfa drugs, and some antibiotics. This condition is more common among people of African, Mediterranean, Middle Eastern, and Southeast Asian descent [19]. These treatments can be both beneficial and dangerous. Without having diverse populations in research, these heterogeneous effects cannot be identified. Standard statistical practice includes methods to *explicitly evaluate the presence of heterogeneity* such as stratified analyses, interaction terms, or multilevel models [15,16].

## Mitigating statistical bias due to external factors

Often there are variables – associated with both outcome and exposure or treatment of interest – that can mask or distort our ability to measure the true relationship of interest. Such variables are referred to as confounders. Including groups that reflect a wide range of characteristics helps distinguish the treatment effect from the influence of potential confounders. For example, if younger individuals are more likely to receive a particular treatment and also tend to have better outcomes regardless of treatment, age may confound the association – making the treatment appear more effective than it truly is. Broad representation in clinical research allows assessment of differences in estimates of the effects of interest, and valid statistical adjustment, that would be missed in homogeneous study samples.

## Conclusion

As scientists collaborate, we must emphasize the basic principles necessary to ensure rigor, validity, and generalizability of study results. Collaborating with institutional officials for legal and ethical guidance and documenting these efforts transparently in protocols and grant applications is recommended to further reinforce a commitment to scientific rigor and research integrity. Broad representation must be viewed as necessary to support scientific goals such as attenuating statistical bias, improving validity and generalizability, promoting efficiency and precision in estimation, and empowering appropriate clinical care.
